# A new reference for changes in X-ray scattering from water due to temperature changes at ambient conditions

**DOI:** 10.1107/S160057752600250X

**Published:** 2026-04-14

**Authors:** Lise G. Hanson, Thomas Veile, Jonathan F. Schouenborg, Victor M. Nielsen, Mads R. V. Jørgensen, Frederik H. Gjørup, Cathrine Frandsen, Kristoffer Haldrup

**Affiliations:** a Department of Physics Technical University of Denmark 2800Kongens Lyngby Denmark; b Department of Chemistry and iNANO, Aarhus University, Langelandsgade, 8000Aarhus, Denmark; c MAX IV Laboratory Lund University Fotongatan 2 221 00Lund Sweden; European XFEL, Germany

**Keywords:** X-ray, scattering, water, solvent response, temperature response

## Abstract

The X-ray solution scattering from water *S*(*Q*)_H2O_ depends on temperature and density, a phenomenon that is utilized in, for example, temperature-resolved and ultrafast time-resolved X-ray solution scattering experiments. Whether this contribution to the acquired data arising from changes in scattering from water is merely a background signal to be subtracted or the primary subject of investigation, reference measurements of the differential changes ∂*S*(*Q*)/∂*T*|_ρ_ are essential. This work presents a newly measured reference dataset for the temperature differential scattering signal of water, acquired at the DanMAX beamline of the MAX IV synchrotron. We compare the new data set to previously archived reference signals and find that analysis based on the older reference may overestimate temperature changes in water by 30–40%. We further demonstrate how the difference scattering signal can be considered linear with temperature up to about a 10 K temperature increase. For larger temperature changes, a second-order contribution d^2^*S*(*Q*)/d*T*^2^ should be taken into account. The new reference datasets and the analysis code are made publicly available for community use.

## Abstract 2

A newly measured reference dataset for the temperature differential scattering signal of water, acquired at the DanMAX beamline of the MAX IV synchrotron, is presented.

## 1. Introduction

X-ray scattering from samples in liquid solution is a powerful technique for obtaining structural information about the sample of interest. Such samples can be either the liquid itself or some chemical species in solution. In the case of the former, the structural properties of water have been studied intensively for many years due to both its environmental relevance and intrinsically interesting properties and structural peculiarities (Neuefeind *et al.*, 2011 ▸; Skinner *et al.*, 2013 ▸; Gallo *et al.*, 2016 ▸; Li *et al.*, 2024 ▸), as recently reviewed by Finney (2024 ▸). Also in the latter case, where the solvent is merely the host medium, water is of particular interest due to its ubiquity in chemistry and, in particular, biochemistry. In many such studies based on X-rays, the sample response to some external or internal stimuli is investigated, and in most, if not all, the processes studied will lead to changes in the temperature of the host medium. The temperature change in turn leads to modifications of the structure of the liquid, leading to changes in the X-ray scattering patterns, which will have to be subtracted or included in subsequent analysis. In order to do so, reference data need to be either readily available or included in the experimental protocol. This is, for instance, relevant to time-resolved ultrafast studies with samples in water and such studies are the main intended application for this article. This study aims to provide a reference for the change in X-ray scattering arising from temperature changes of water, ∂*S*(*Q*)/∂*T*|_ρ_. The reference is determined for ambient conditions and with intermediate *Q*-range (*Q* = 0.6–4.5 Å^−1^) where *Q* is the length of the scattering vector [*Q* = (4π/λ)sin(2θ/2)]. We compare the new results with the existing reference data and further investigate the linearity of this response.

### 1.1. Background

Time-resolved X-ray solution scattering (TR-XSS) in the ultrafast (*t* < µs) time regime was primarily developed at the ID09B beamline of the ESRF synchrotron in the early 2000s (Plech *et al.*, 2004 ▸). In the following years, the technique spread to other synchrotron facilities (Nozawa *et al.*, 2007 ▸; Graber *et al.*, 2011 ▸) and the theoretical foundations for understanding the solvent’s response to ultrafast (‘impulsive’) laser heating were laid down (Cammarata *et al.*, 2006 ▸). Most often in TR-XSS studies, the starting point for analysis is the difference signal Δ*S*(*Q*, *t*) = *S*(*Q*, *t*)_laser-on_ − *S*(*Q*)_laser-off_ as the difference signal isolates the changes in scattering arising due to laser excitation at *t* = 0. Thus, the difference scattering significantly suppresses the background contributions arising from, for example, air or slit scattering. The seminal work by Cammarata *et al.* demonstrated that the time-dependent changes in the difference signal Δ*S*(*Q*, *t*) arising as a consequence of impulsive heating of the solvent could be described through a linear combination of two differential scattering terms as 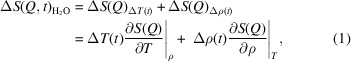
where *T* denotes the solvent temperature and ρ is the density. The partial derivatives are taken at constant density and temperature, respectively. The study of Cammarata *et al.* also provided a procedure where a series of steady-state measurements of *S*(*Q*, *T*) at a range of temperatures were complemented by a measurement utilizing impulsive IR excitation and sub-nanosecond time resolution to heat the sample and record the resulting changes in scattering under isochoric (constant-ρ) conditions. This combination of measurements was demonstrated to be able to provide the Δ*T* and Δρ differentials for any given solvent. The characteristic time scales for the dynamics of these two signal components were determined to be sub-100 ps and tens of nanoseconds, respectively. Very recently similar experimental approaches (impulsive IR laser heating) have been utilized in the deeply super-cooled regime to investigate fundamental structural properties of water as well (Pathak *et al.*, 2021 ▸; Tyburski *et al.*, 2026 ▸).

**Figure 1:**
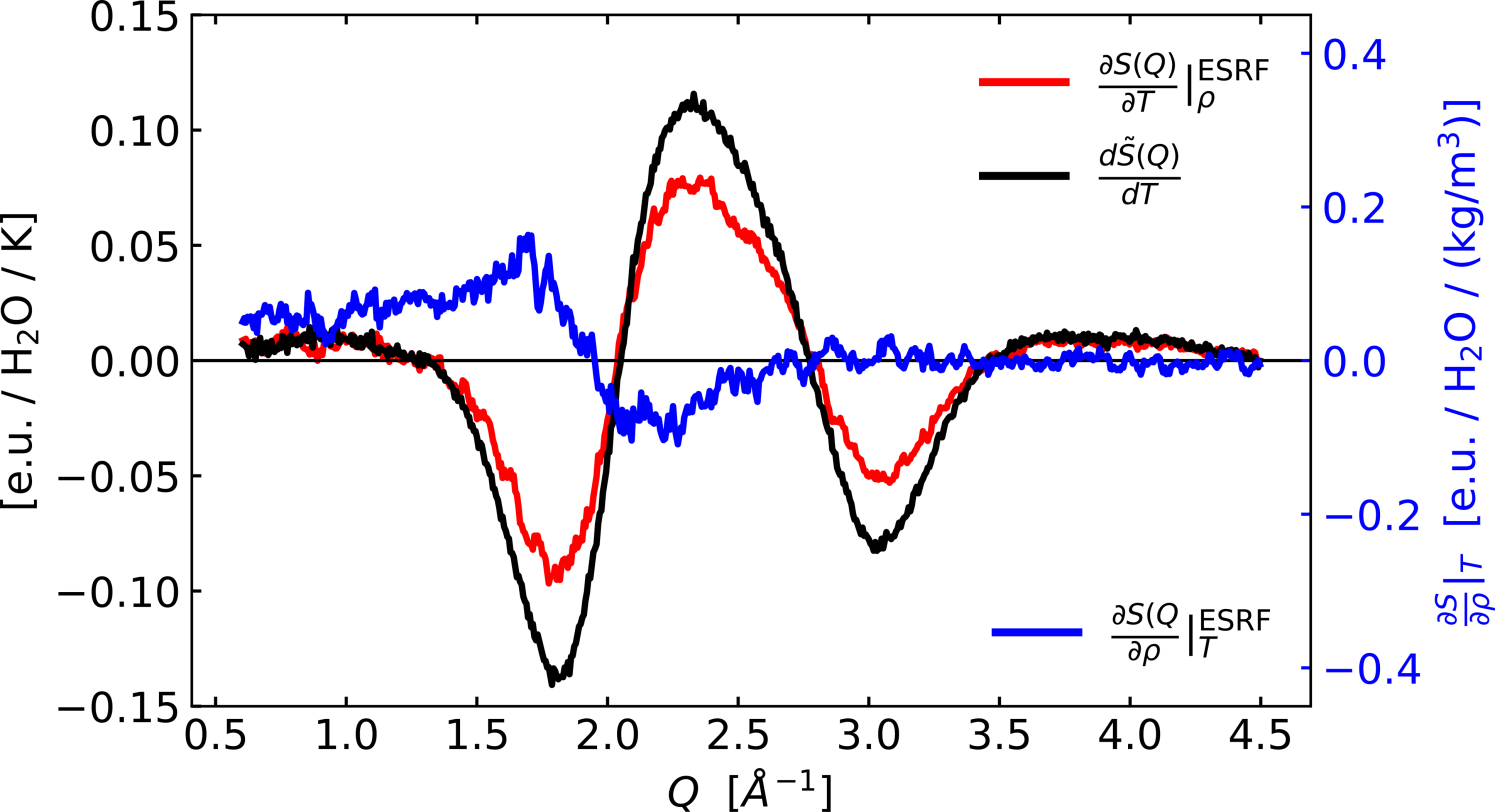
Differential reference signals measured at ESRF at ID09B on water by Kjær *et al.* (2013 ▸) (red and blue) compared with the newer 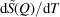 reference signal measured at DanMAX (black). Very similar shapes, but a significant difference in the magnitude of the ∂*S*(*Q*)/∂*T*|_ρ_ signals are notable.

Building on the work by Cammarata *et al.*, Kjær and co-workers (Kjær *et al.*, 2013 ▸) established how laser excitation of a structurally inert dye could also be utilized for the impulsive heating of the solvent. Via this method the Δ*T* and Δρ differentials were determined for a range of organic and inorganic solvents and presented as archived data (repository Kjær *et al.*, 2013 ▸). Notably, the suitability of specific dyes for providing this information for a range of solvents, including water, was discussed in that work, but the corresponding differential scattering signals for water were not included or discussed in the original publication. Still, a somewhat noisy data set was acquired and archived in the same data repository and subsequently used in other works (Canton *et al.*, 2015 ▸; Haldrup *et al.*, 2016 ▸; Kjær *et al.*, 2019 ▸). Fig. 1 ▸ shows the differential changes 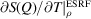 and 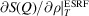 as acquired for water at the ID09B beamline at the ESRF facility (Kjær *et al.*, 2013 ▸) and compares these with the new reference signal 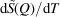 proposed in this study. More recently, acquisition of Δ*S*(*Q*)_Δ*T*_ has been integrated into the experimental protocols in ultrafast solution-state X-ray studies (Khakhulin *et al.*, 2019 ▸; Kabanova *et al.*, 2024 ▸) with either molecular dynamics (MD) simulations or the original reference data archived by Kjær *et al.* used to scale the acquired difference signal.

### 1.2. Analysis approaches

From a data analysis point of view, two main approaches have been taken in the literature on solution-state structural dynamics studied with TR-XSS. In the first, the contribution to the acquired difference scattering signals arising from the heating and hydrodynamic evolution of the solvent has been considered merely a background to be removed. This can be challenging, as the total difference signal in such studies is very often <1% of the total scattering signal, and the contribution to the difference signal from the solvent may be five to ten times larger than the solute-related difference signal of interest (Markmann *et al.*, 2024 ▸). This requires careful scaling and fitting methods, and robust and powerful algebraic approaches [known as SANOD (Ki *et al.*, 2019 ▸) and PEPC (Ki *et al.*, 2023 ▸)] have also been developed towards this goal.

In the second approach, and of direct relevance to the present work, the magnitude of the solvent-specific contribution to the difference scattering signal is of importance for the analysis and interpretation of the acquired data via calorimetry, where the energy release to the solvent is estimated from the temperature increase determined from the changes in X-ray scattering. For such studies, there is a natural need to calibrate the change in solvent X-ray scattering to a known reference. Alternatively, the needed reference data may also be acquired by using a temperature-controlled heat bath for the sample reservoir, although it may be challenging to ensure similar temperatures in the reservoir and at the beam/sample interaction point. Examples of such ultrafast calorimetry studies include both what is known as *T*-jump approaches with direct solvent excitation as well as studies where the energy release to the solvent following photo-excitation is of interest. The former case of ultrafast *T*-jump studies involve for instance near-infrared (NIR) excitation of the solvent to induce a temperature increase which in turns lead to, for example, dissociation of insulin *N*-mers (*N* = 2–6) (Rimmerman *et al.*, 2017 ▸; Rimmerman *et al.*, 2018 ▸), unfolding of proteins [*e.g.* apomyoglobin (Henry *et al.*, 2020 ▸)], or enzyme activation [cyclophilin A (Thompson *et al.*, 2019 ▸)].

The experimental challenges are more complex for studies addressing the magnitudes and time scales of the energy release from a photoexcited solute to the surrounding solvent medium (Leshchev *et al.*, 2018 ▸). In many cases, the relevant time scales entail that the solvent heating takes place under isochoric conditions, precluding the direct use of steady-state reference measurements with a heat bath. For quantitative analysis, this necessitates using a known, scaled reference signal ∂*S*(*Q*)/∂*T*|_ρ_ obtained with the methods outlined by Cammarata *et al.* and Kjær *et al.* as introduced above. As discussed in these method papers, this scaling is challenging. Additionally, several recent studies have found disagreement where the estimated temperature increase of the solvent has been reported to be higher than expected from the independently determined excitation fractions and differences in energies between the relevant excited states (Haldrup *et al.*, 2016 ▸; Leshchev *et al.*, 2018 ▸; Kabanova *et al.*, 2024 ▸; Hansen *et al.*, 2025 ▸). Other studies have found better agreement (Haldrup *et al.*, 2012 ▸; Khakhulin *et al.*, 2019 ▸; Biasin *et al.*, 2022 ▸), but it is at present unclear whether the better agreement in these studies arises from differences in experimental methodology or the use and scaling of the solvent reference signal(s).

On this background, we identify a clear need for an updated reference for the difference X-ray scattering signal arising as a consequence of a temperature increase of pure water. In this work we describe how we have determined the ∂*S*(*Q*)/∂*T*|_ρ_ difference scattering signal using external heating of a water sample utilizing quantitative subtraction of the previously determined isothermal density difference signal 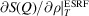 from the acquired data. A comparison of the difference signal shape with the archived reference data yields a high degree of similarity after correction for X-ray bandwidth differences, but the magnitude of the newly determined ∂*S*(*Q*)/∂*T*|_ρ_ is found to be significantly different from the previously archived reference data. The analysis is further expanded to include a discussion of higher-order contributions to the difference signal in line with what has previously been reported for super-cooled water (Neuefeind *et al.*, 2011 ▸). The new reference data sets have been archived along with the data reduction and data analysis code.

## 2. Methods

### 2.1. Experimental setup

**Figure 2:**
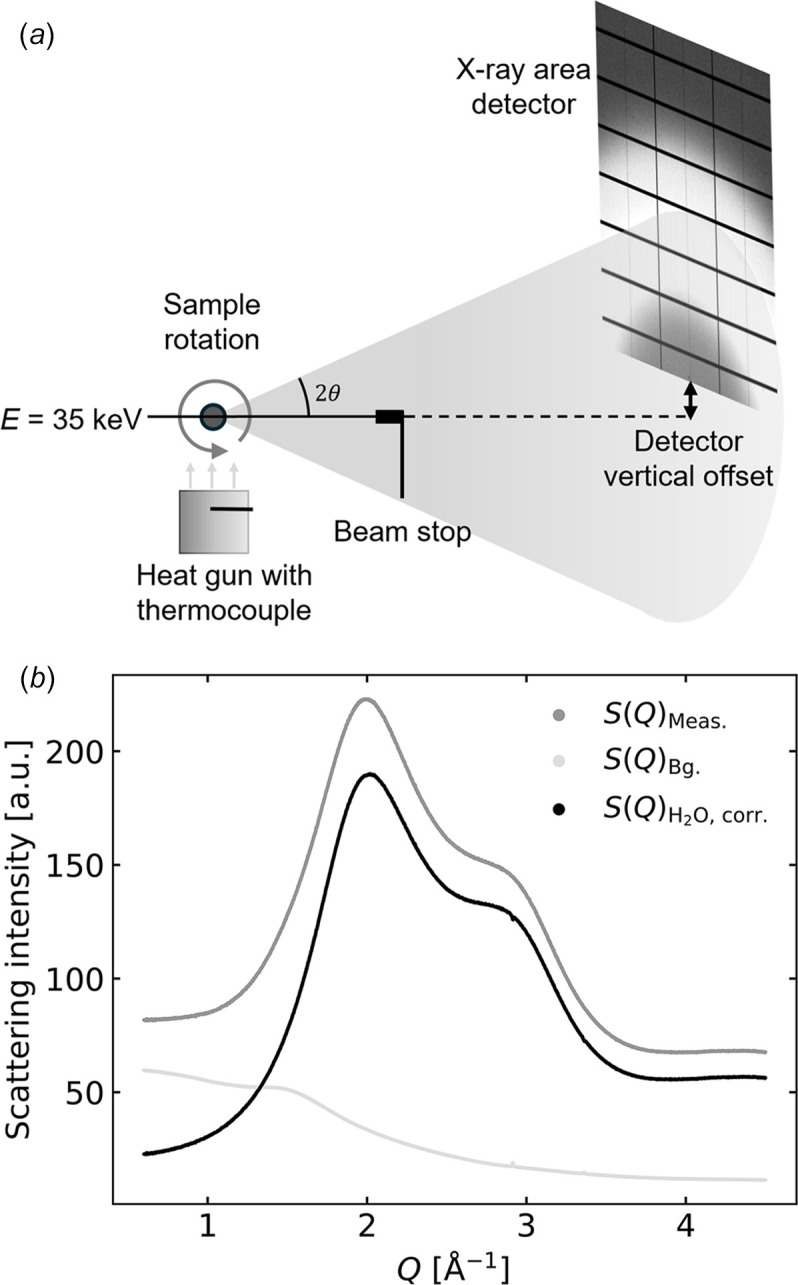
(*a*) Experimental setup: a 0.7 mm × 0.9 mm 35 keV X-ray beam hits the rotating sample capillary (2 mm) with the scattered X-rays detected by a vertically offset area detector at a sample–detector distance of 1240 mm. Sample temperature is controlled by a heat gun. (*b*) Azimuthally integrated X-ray scattering signals from a water-filled capillary, *S*(*Q*)_Meas._ (dark grey) at 300 K, an empty capillary *S*(*Q*)_Bg._ (light grey), and the background-corrected scattering signal 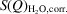 (black) from water.

Fig. 2 ▸(*a*) illustrates the experimental setup used for the study presented here, which was carried out at the DanMAX beamline of the MAX IV synchrotron facility. The X-ray beam was generated by an in-vacuum undulator and monochromated to 35 keV by a Si(111) double-bounce monochromator. The beam was focused to 890 µm × 740 µm (full width at half-maximum) at the sample position. The Milli-Q water sample was mounted in a thin-walled borosilicate glass capillary (diameter 2 mm, wall thickness 10 µm) and sealed by hot glue. The sample capillary was mounted on a spinner directly above a heat gun (HyBec) used to control the sample temperature. The sample temperature is determined from a calibration measurement relating the temperature measured in the heat gun nozzle to the temperature measured at the sample position when substituting the sample with a thermocouple. This procedure is described in detail in Section SI7 of the supporting information, and indicates a confidence interval on the sample temperature of 0.07 K. Scattered X-rays were detected by a Dectris PILATUS3 X 2M CdTe detector placed at a sample distance of 1240 mm and vertically offset by 180 mm from the beam to cover a larger *Q*-range. The sample–detector distance was calibrated with an LaB_6_ reference (NIST SRM660c), and the 2D scattering images were subsequently corrected for polarization and geometry effects using *PyFAI*-based (ESRF, 2025 ▸) software with subsequent azimuthal integration carried out using in-house DanMAX software based on algorithms as described by Jensen *et al.* (2022 ▸). This yielded *S*(*Q*) data sets with a *Q*-axis bin size of 0.0014 Å^−1^, but for ease of comparison with existing reference data sets (Kjær *et al.*, 2013 ▸) the *S*(*Q*) data acquired at DanMAX were linearly interpolated onto the coarser *Q*-axis of the reference data sets, having a bin size of 0.006 Å^−1^. The linear interpolation was performed after azimuthal integration. Fig. S1 of the supporting information shows how the *S*(*Q*) data acquired at DanMAX are in very good agreement with the previous high-resolution study by Skinner *et al.* (2013 ▸).

### 2.2. Data acquisition, background correction and quantitative scaling

For the measurements described here, scattering data *S*(*Q*) were acquired as a series of *N* ten-second data acquisitions while the temperature was ramped from 300 K to 330 K at a rate of 3 K min^−1^, yielding a temperature-resolved data series with a step size of 0.5 K. The temperature scan was preceded and followed by 60 and 120 s of steady-state measurements at constant *T*_Start_ = 300 K and *T*_Finish_ = 330 K. To account for changes in air and capillary background scattering, the scattering from an empty capillary was also acquired as a function of temperature in a separate experiment, see Section SI2.

The scan series acquired as described above yields a set of *NS*(*Q*)_Meas._ curves, and to account for slight changes in incident intensity these were individually normalized to an upstream *I*_0_ monitor. Following this initial step, the scattering data*S*(*Q*)_Meas._ were assumed to be given by a combination of the very well known scattering from pure water 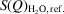 (Skinner *et al.*, 2013 ▸) and the background (air and capillary scattering) *S*(*Q*)_Bg._, *i.e.*
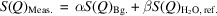
where α and β are scaling constants to be determined in a fit. From the *S*(*Q*)_Meas._ curves, we define the background-corrected scattering from pure water as 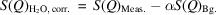
with α determined from an average of *S*(*Q*)_Meas._ during steady-state conditions at 300 K. The temperature 300 K was chosen as this was the closest to the temperature at which the reference signal 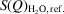 was acquired, namely 295 K. Due to the heating-only nature of the experimental setup, 300 K was the lowest reliable temperature for which data were acquired. Fig. 2 ▸(*b*) shows the measured data, background, and corrected data. Finally, these background-corrected 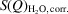 curves were each scaled to the water reference data introduced above (Skinner *et al.*, 2013 ▸) to yield 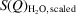 in electron units per water molecule (e.u./H_2_O). In the following, we define *S*(*Q*) = 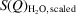 for clarity of presentation and provide an investigation of how this signal changes as a function of temperature. Below, a shape-preserving Savitzky–Golay filter has been used for smoothing the data when relevant. When applied, the filter always has a polynomial order of three and a length of 0.5 Å^−1^.

## 3. Results

**Figure 3:**
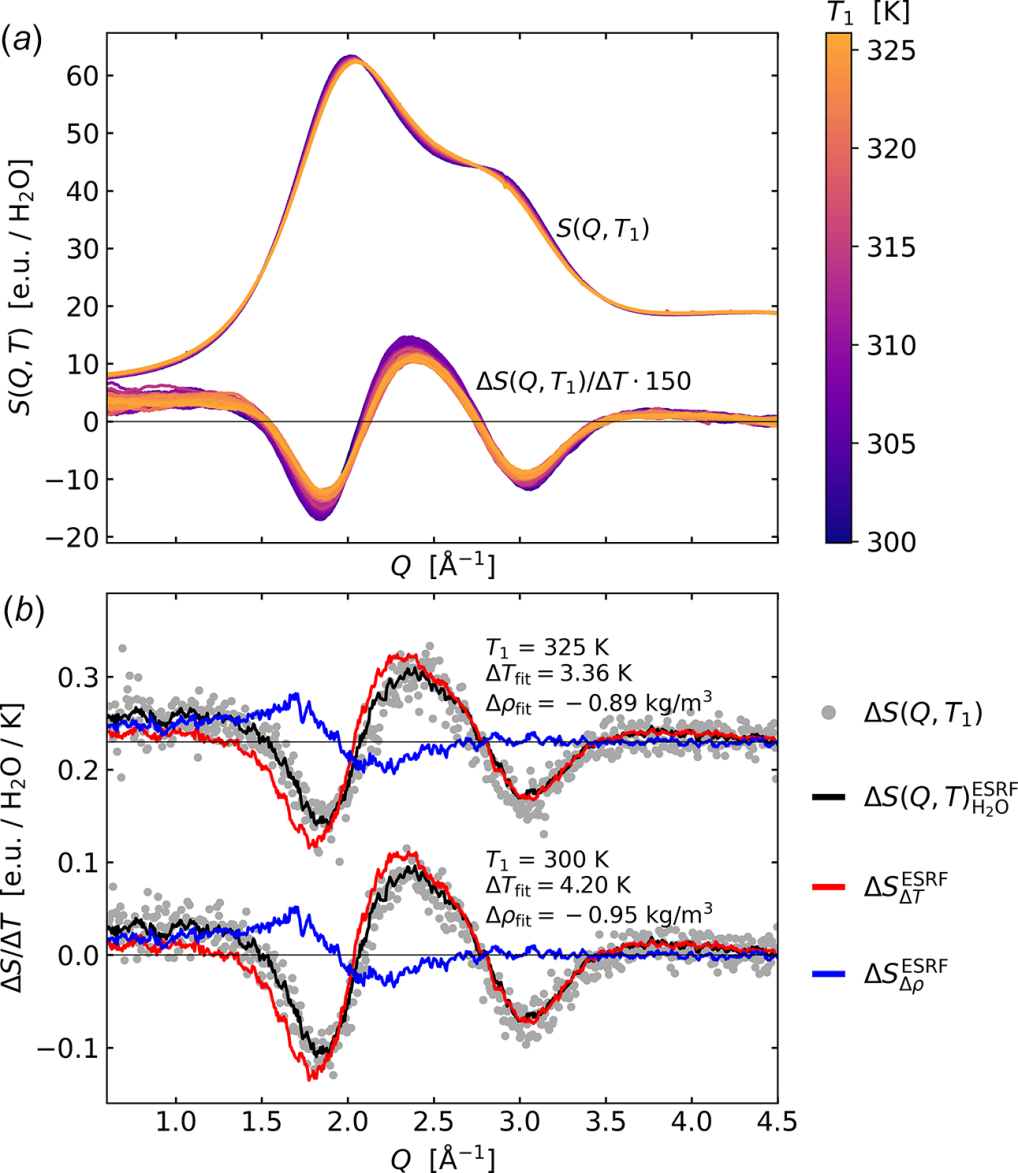
(*a*) *S*(*Q*, *T*) and corresponding normalized difference signals Δ*S*(*Q*, *T*_1_)/Δ*T* for *T*_1_ = 300–325 K and Δ*T* = 3 K, colour-coded according to *T*_1_. The difference data are smoothed for clarity of presentation. (*b*) Δ*T* = 3 K difference signals for *T*_1_ = 300 K and 325 K (grey circles, 325 K data offset) with a fit (black line) of the ESRF difference scattering signal 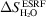, which is a linear combination of 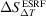 (red) and 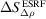 (blue) as defined in equation (1) (Kjær *et al.*, 2013 ▸). Best-fit results for Δ*T*_fit_ and Δρ_fit_ are indicated in the legends.

Fig. 3 ▸(*a*) shows the set of acquired scattering signals as a function of acquisition temperature, *S*(*Q*, *T*), with a bin size of 0.5 K and colour-coded according to temperature. Highlighting the small changes in signal shape, the same panel also shows the normalized difference scattering signal defined as Δ*S*(*Q*, *T*_1_)/Δ*T* = [*S*(*Q*, *T*_1_ + Δ*T*) − *S*(*Q*, *T*_1_)]/Δ*T* and where *T*_1_ varies from *T* = 300 K to 325 K and Δ*T* = 3 K. The normalized difference signal is multiplied by a factor of 150 and colour-coded according to *T*_1_. It is evident that, while the temperature-dependent changes to *S*(*Q*) are small (about 0.1% of the total signal per degree K), they show a high degree of similarity in (difference) signal shape as a function of *T*_1_. Still, some tendency towards decreased (difference) signal magnitude as a function of *T*_1_ can be seen. The difference signals have been smoothed with the above-mentioned Savitzky–Golay filter.

Fig. 3 ▸(*b*) shows two of the difference signals (grey circles) from the full set shown in panel (*a*) overlaid with a fit (black line) of the expression presented in equation (1). The 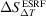 and 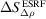 fit-components (old ESRF reference) are shown with red and blue lines, respectively. The fitted reference measurements are based on ultrafast excitation of a structurally inert dye measured at ESRF as described in detail by Kjær *et al.* (2013 ▸). While the fit quality is very good, it is noticeable that the best-fit value Δ*T*_fit_ for the two different *T*_1_ with the same nominal Δ*T* = 3 K are found to be 4.20 ± 0.05 K and 3.36 ± 0.05 K. Thus, both Δ*T*_fit_ values are significantly larger than the 3 K expected. The value found for Δρ_fit_ = −0.95 ± 0.04 kg m^−3^ (∼0.1% density change) is in good agreement with the expected density decrease due to thermal expansion from tabulated values (Lemmon, 2010 ▸), whereas Δρ_fit_ = −0.89 ± 0.04 kg m^−3^ is less than the expected value of −1.4 kg m^−3^.

**Figure 4:**
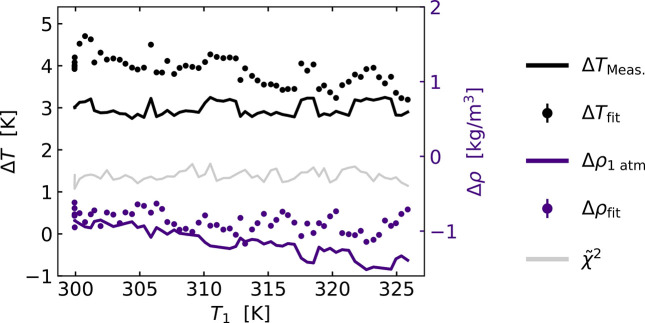
Fit parameters Δ*T* (left axis) and Δρ (right axis) as well as 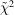 (left axis) along the temperature ramp of *T*_1_. The fits are calculated using Δ*T* = 3 K and from fitting Δ*S*(*Q*, *T*) with the ESRF reference signals as seen in Fig. 3 ▸. Δ*T*_Meas._ and the expected values Δρ_1atm_ are shown for comparison. Δρ_1atm_ is calculated based on tabulated water density data at a pressure of 1 atm (Lemmon, 2010 ▸) and the measured temperature change, Δ*T*_Meas._.

To quantify these observations further, Fig. 4 ▸ shows the best-fit results for Δ*T*_fit_ and Δρ_fit_ as a function of *T*_1_ along the temperature scan. Fig. 4 ▸ also reports the fit quality for each value of *T*_1_ in terms of the reduced 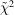. In agreement with visual inspection of the difference signals shown in Fig. 3 ▸, Δ*T*_fit_(*T*_1_) shows a slightly decreasing trend along *T*_1_, although consistently higher than the expected Δ*T* = 3 K. Δρ_fit_(*T*_1_) is near-constant as a function of time, although the tabulated values suggest that a decreasing value for this parameter should be evident (Lemmon, 2010 ▸). No change in fit quality (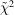) is seen as a function of *T*_1_.

**Figure 5:**
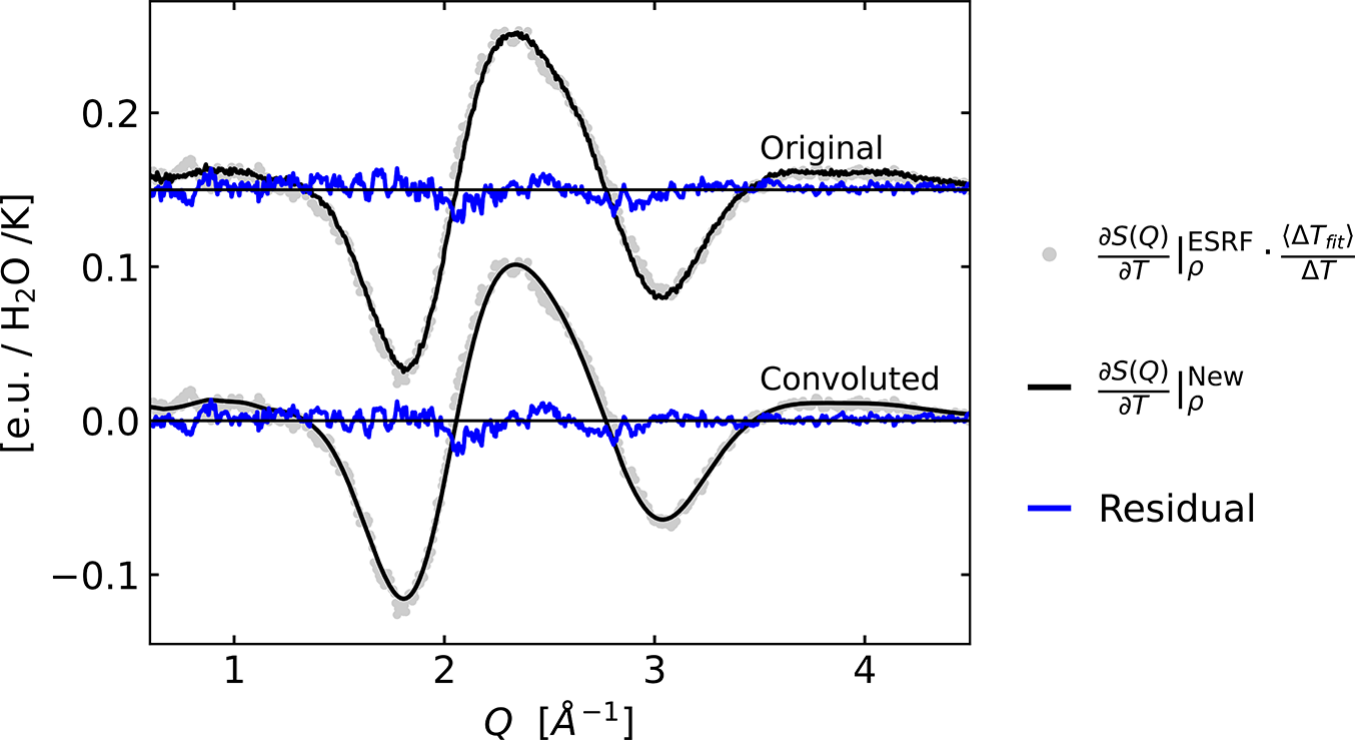
Comparison of difference signals 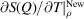 acquired at DanMAX (Δ*T* = 3 K) with the reference signal 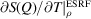. The 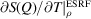 curve is scaled by 〈Δ*T*_fit_〉/Δ*T* to match the scaling of the two signals, allowing for a comparison of the shape of the two signals. The lower plot includes a convolution of 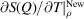 to account for differences in X-ray energy bandwidth. An offset is used for clarity.

The fit approach described above allows isolation of the isochoric changes in X-ray scattering arising from a temperature change (Δ*T* = 3 K) only as 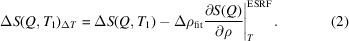
Δρ_fit_ was determined via least-squares fitting as described in connection with Figs. 3 ▸ and 4 ▸ above. Before subtraction, 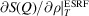 was smoothed (Savitzky–Golay filter) to avoid introducing noise from the rather low signal-to-noise ratio for the tabulated reference data. Based on this density contribution subtraction and averaging (〈…〉) over all *T*_1_ a new reference is proposed from the DanMAX data as 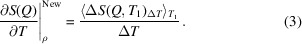
Fig. 5 ▸ shows a comparison between 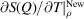 calculated from the mean of the set of density-subtracted difference signals and the older reference differential 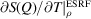 acquired via dye-mediated solvent heating at ESRF. The latter is scaled by 〈Δ*T*_fit_〉/Δ*T* to match the scaling of the two signals, allowing for a more direct comparison of their shapes. Visual inspection shows that the 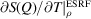 curve is slightly broadened along *Q* due to the ∼Δ*E*/*E* = 2.5% bandwidth of the multilayer-monochromated beam employed to acquire this older data set. Fig. 5 ▸ also shows the spectrally broadened version of the difference signal acquired at DanMAX adapted to the ESRF reference by convoluting the acquired data with a Gaussian function in combination with a slight vertical offset and magnitude rescaling. Best agreement is found for a Δ*E*/*E* value of 2.9%, slightly higher than the 2.5% bandwidth reported in the work of Kjær *et al.* for the ESRF reference.

**Figure 6:**
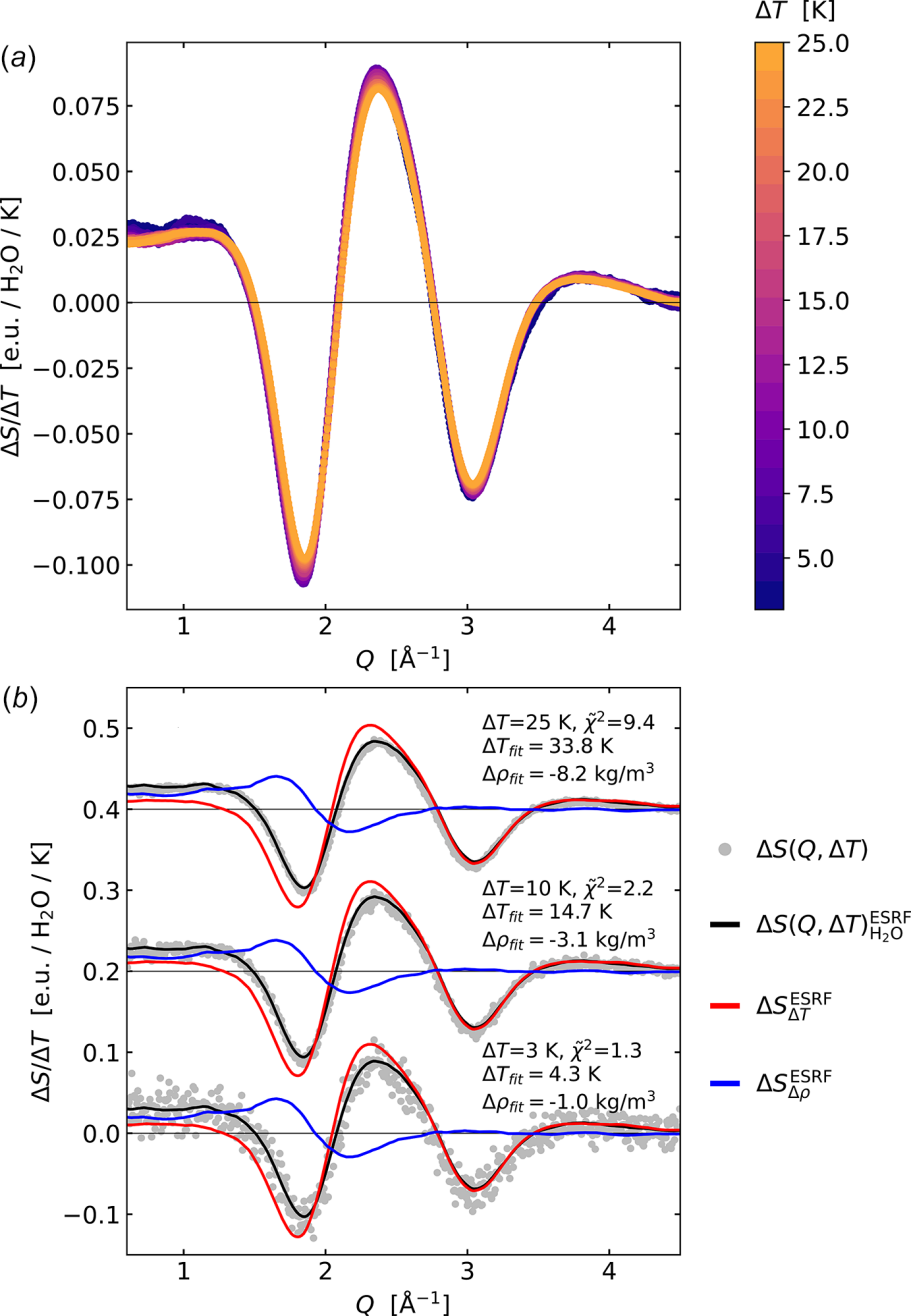
(*a*) Difference signals colour-coded according to their value of Δ*T* in a range from 3 to 25 K. The data are smoothed with a Savitzky–Golay filter for clarity. (*b*) Difference signals, fits and residuals for Δ*T* = 3, 10, and 25 K, *T*_1_ = 300 K. Grey circles indicate measured data points, the black line is the fit of the ESRF difference scattering signals, red is 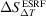 and blue is 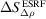. 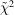 and best-fit results for Δ*T*_fit_ and Δρ_fit_ are indicated in the legends.

We now turn to the question of whether Δ*S*_Δ*T*_ can be considered linear with Δ*T*. The results presented in Figs. 3 ▸ and 4 ▸ indicate that the implicit assumption of 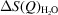 = Δ*T*[∂*S*(*Q*)/∂*T*]|_ρ_ + Δρ[∂*S*(*Q*)/∂ρ]|_*T*_ being linear in Δ*T* is not valid across the entire temperature region from 300 to 326 K due to the decrease of Δ*T*_fit_ from around 4 K to 3.5 K, relative to the measured value of 3 K. Highlighting this observation further, Fig. 6 ▸(*a*) shows Δ*S*/Δ*T* calculated for Δ*T* varying from 3 to 25 K with *T*_1_ fixed to 300 K. The shape and height of Δ*S*/Δ*T* is observed to change depending on Δ*T*, which originates from the contribution from Δ*S*_Δρ_ and a contribution not captured by the linear model in equation (1) when using the ESRF reference data from Kjær *et al.* (2013 ▸) as discussed in further detail below.

Fig. 6 ▸(*b*) shows the same data representation and analysis as in Fig. 3 ▸(*b*) applied for difference signals acquired with Δ*T* = 3 K, 10 K, and 25 K. From the increase in 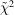 values, a clear tendency towards worse fits for larger Δ*T* is evident. We note that in this analysis the ESRF difference signals, which constitute the fit components, have been smoothed with the Savitzky–Golay filter mentioned above in order to avoid introducing noise via the increasing magnitude of the (somewhat noisy) fit components. The differences in relative noise level for Δ*S*(*Q*, Δ*T* = 3, 10, 25 K) are explicitly included in the analysis via the definition of 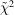 and, as such, the observed increase in 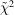 with increasing Δ*T* can therefore be directly attributed to a worse fit, and not to differences in relative noise in the data nor in the fit components with increasing Δ*T*.

**Figure 7:**
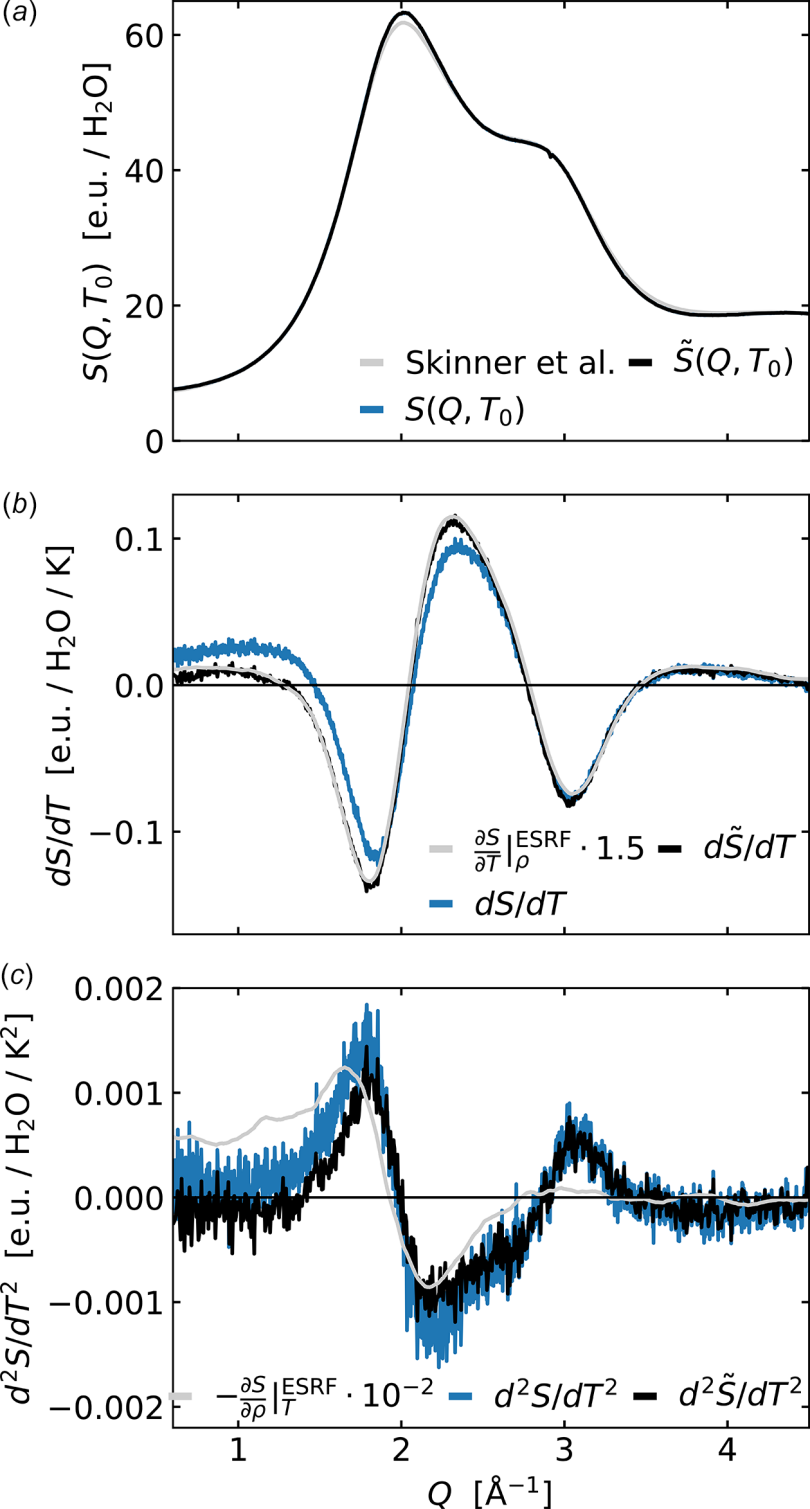
*Q*-resolved Taylor expansion of *S*(*Q*, *T*) (blue) and 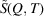 (black). For the data shown in black, the density contribution to *S*(*Q*) has been subtracted before the Taylor analysis as defined in equation (5). (*a*) Zeroth components *S*(*Q*, *T*_0_) and the *S*(*Q*) reference data determined by Skinner *et al.* (grey). (*b*) First component d*S*(*Q*)/d*T* and the temperature differential determined at ESRF (grey). (*c*) Second component d^2^*S*(*Q*)/d*T*^2^ and the density differential determined at ESRF (grey). The ESRF reference signals are multiplied by a factor of 1.5 and −10^−2^, respectively, to plot them on a similar scale to the Taylor coefficients. Additionally, they are Savitzky–Golay filtered, as also applied in the signal fitting and subtraction of 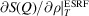.

The significantly worse fits for Δ*T* = 10 K and 25 K compared with the fits for Δ*T* = 3 K indicate that the assumption of signal linearity may only hold for rather small Δ*T*, as also established for X-ray studies of supercooled water (Neuefeind *et al.*, 2011 ▸; Benmore *et al.*, 2019 ▸; Esmaeildoost *et al.*, 2021 ▸). To investigate this deviation from the linear model further, we follow the approach of Neuefeind *et al.* (2011 ▸) where a second-order Taylor expansion along *T* is taken for each *Q*-point in the *S*(*Q*, *T*) data series, *i.e.*
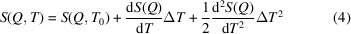
where *T*_0_ is the baseline temperature, here 300 K, and Δ*T* =*T* − *T*_0_. Expanding on the previous work of Neuefeind *et al.* (2011 ▸), we here apply the Taylor expansion analysis to both *S*(*Q*) as well as to 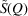 defined as 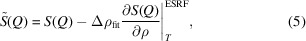
where the density-change contribution to the scattering signal is fitted and subtracted for each temperature step before applying the Taylor expansion analysis. Fig. 7 ▸ shows the result of both analysis approaches.

From Fig. 7 ▸(*a*), the agreement between the high-quality reference scattering data from Skinner *et al.* (2013 ▸) (grey line) and the zeroth-order contribution to the DanMAX data set (black line) is very good. Fig. 7 ▸(*b*) illustrates how subtracting the density contribution to the set of scattering data before the Taylor analysis leads to a first-order contribution (black line) very similar in shape to the solvent heating differential previously determined at ESRF (grey line) and from the DanMAX data sets as described above. The blue data trace in the middle panel demonstrates how *not* subtracting the density contribution prior to the Taylor analysis leads to a positive feature in the difference signal at low *Q* not observed for the temperature differential as acquired at ESRF. Fig. 7 ▸(*c*) shows how the second-order contribution (black and blue lines) to the changes in X-ray scattering with temperature is highly similar, but not identical, to the density differential 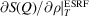, which is shown in grey. It is noticeable that the second-order contribution is less sensitive to whether or not the density contribution has been removed prior to the Taylor expansion analysis. Quantitative comparison with the previous Taylor expansion analysis results reported by Neuefeind *et al.* (2011 ▸) is difficult due to the very different *Q*-axis and data representations applied, but there is good general agreement between the signal shapes in the *Q* = 1.5–4 Å^−1^ region. However, the low-*Q* negative feature seen in the data set and analysis reported by Neuefeind *et al.* is not reproduced by the present analysis. We finally note that for a temperature increase of Δ*T* = 3 K the positive features of the first- and second-order terms have a ratio of [0.1 e.u. K^−1^ 3 K] / [(1/2)0.0015 e.u. K^−2^ (3 K)^2^] ≃ 40 whereas for Δ*T* = 30 K this ratio becomes ∼4. This comparison of the magnitudes of the first- and second-order contributions is thus useful to estimate whether linearity can be assumed in equation (1). It is noticed that the existence of non-linearities with Δ*T* and Δρ introduces a need for using a reference signal [∂*S*(*Q*)/∂*T*|_ρ_ and ∂*S*(*Q*)/∂ρ|_*T*_] measured at the temperatures of interest and that this reference should only be used within a rather narrow temperature interval. In the next section, we briefly discuss the underlying structural changes in water that give rise to the first- and second-order difference signals.

## 4. Discussion and conclusions

As shown by Fig. 7 ▸ and Fig. S1 of the supporting information, the X-ray scattering data from water acquired at the DanMAX beamline at constant temperature and following subtraction of background scattering contributions is in very good agreement with previous results reported by other groups using somewhat more involved methods of data acquisition and background subtractions (Skinner *et al.*, 2013 ▸). Fig. 3 ▸(*a*) shows how this scattering pattern changes as the sample temperature increases, with the difference scattering signal Δ*S*(*Q*, *T*) exhibiting a distinctive oscillatory pattern. These changes arise from the lowering of short-range order with temperature and the associated changes in local packing of the water molecules as described and discussed in detail in previous work utilizing steady-state data acquisition with a large *Q*-range (Skinner *et al.*, 2014 ▸). Fig. 3 ▸(*b*) shows how Δ*S*(*Q*, *T*_1_, Δ*T* = 3K) is very well captured by a linear combination of the two solvent differentials determined and utilized in previous ultrafast studies of structural dynamics in liquid solution. In terms of the underlying structural changes giving rise to the changes in the shape of the X-ray scattering patterns, the density differential ∂*S*/∂ρ|_*T*_ has a straightforward interpretation as arising from the mean distance between molecules becoming slightly longer, leading to a shift of the characteristic peak in *S*(*Q*) towards lower *Q*. Interpretation of ∂*S*/∂*T*|_ρ_ is more involved as discussed in the aforementioned article by Skinner *et al.* and related works (Skinner *et al.*, 2014 ▸; Pathak *et al.*, 2019 ▸; Benmore *et al.*, 2019 ▸) and further outlined below.

Of particular interest to previous and future studies on ultrafast time scales, the quantitative scaling constants Δ*T* and Δρ (Fig. 4 ▸) indicate that the magnitude of 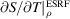 data archived by Kjær *et al.* may have been under-estimated by 30–40%. This discrepancy could potentially arise from the assumption of linearity in the sample response to laser excitation in the original study, which was conducted at fairly high laser fluences where this may not be completely valid. The lower magnitude of the reference signal may in turn lead to higher estimates of energy release to the solvent in the ultrafast experiments discussed in the *Introduction*[Sec sec1], and we note that this may explain some of the too-high energy release estimates in these studies. Still, the analysis results reported in Fig. 5 ▸ demonstrate how the shape of the 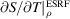 differential is in very good agreement with the density-corrected difference signal obtained in the present study 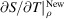, calculated based on a temperature difference of Δ*T* = 3 K, in particular when the spectral broadening of the original reference data set from ESRF is taken into account. This good agreement is a key observation, as an erroneous shape of the difference signal utilized in the previous TR-XSS studies could have impacted the results of the structural refinements of the (excited-state) solute structures that were some of the key objectives of those and similar studies.

Going beyond the temperature changes usually encountered in ultrafast studies, Fig. 6 ▸ shows how the fit quality decreases from 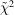 = 1.3 to 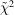 = 9.4 when Δ*T* is increased from 3 K to 25 K. The Taylor expansion analysis presented in Fig. 7 ▸ shows how this likely arises from a departure from the linearity assumed in equation (1) for larger Δ*T*. Interestingly, the second-order contribution to the difference signal arising from a temperature change is quite similar to the solvent differential arising from a change in density, but they are not identical. In particular, the second-order term exhibits a positive signal feature around *Q* = 3 Å^−1^ which is completely absent in the density differential. We also note that the shape of the second-order component is quite similar to what was reported based on MD simulations in the supplementary online information of Haldrup *et al.* (2016 ▸). Still, the similarity in signal shapes between the second-order component and the density differential may be relevant to the discussion of the presence/absence of signal components related to density differences at very short time scales in previous ultrafast studies (Khakhulin *et al.*, 2019 ▸). The non-linearity in Δ*T* observed from the analysis in Figs. 4 ▸ and 7 ▸ entail that the experimentally determined temperature differential signal ∂*S*/∂*T*|_ρ_ for water should only be used in a rather narrow temperature region.

Throughout this study, three different types of first-order derivatives have been in play, namely 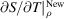, d*S*/d*T*, and 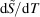. In future studies we recommend using 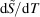 as a new reference in the following way, 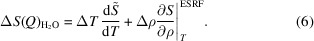
We recommend the use of 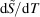 since this quantity is density corrected and approximates the derivative at 300 K most precisely compared with 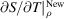 which is most precise at 313 K due to averaging in equation (3). A temperature near 300 K is most relevant, as this is typically used in TR-XSS studies. In principle, 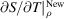 could have been determined closer to 300 K with the data measured at DanMAX, but this would require an average over a smaller region in *T*_1_ and thus result in a low signal-to-noise ratio (see Section SI8). The 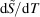 reference can be used up to Δ*T* = 14 K without taking into account the second-order derivative (second-order/first-order < 0.1). The second-order contribution, 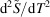, can be used to quantify the error of the linear temperature assumption, or it can be used to evaluate the value of 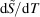 at higher temperatures, *e.g.* 325 K.

From a structural point of view, the temperature dependence of the peak features present in *S*(*Q*) at *Q* = 2 Å^−1^ and at *Q* = 3 Å^−1^ have been studied previously in much detail (Skinner *et al.*, 2014 ▸), and the signal dynamics seen in Fig. 3 ▸(*a*) reproduce the merging of these two features with increasing temperature. This observation has been related to a complicated interplay between temperature-dependent structural changes, which maintain the local tetrahedral coordination of the water molecules while allowing for a general expansion with temperature above *T* = 4°C. This expansion is clearly reflected in the isothermal density differential previously determined at ESRF [see Fig. 3 ▸(*b*) blue line and Fig. 7 ▸(*c*) grey trace line] where the main peak in *S*(*Q*) shifts towards lower *Q* with increasing temperature, *i.e.* corresponding to longer distances in a real-space representation. From the shape of the second-order contribution [Fig. 7 ▸(*c*) black line], one can further observe that the evolution of the peak height of the first feature in *S*(*Q*) (*Q* = 2 Å^−1^) is near-linear in *T* but with significant quadratic contributions to the peak shape. This is in direct contrast to the second peak in *S*(*Q*) (*Q* = 3 Å^−1^), where the evolution of the peak height has significant quadratic dependence on *T*. A full interpretation of these observations is beyond the scope of the present work, but the results and methodology presented here could be of interest to the still-ongoing discussions and debate about the fundamental structural properties of water (Benmore *et al.*, 2019 ▸; Pathak *et al.*, 2019 ▸; Esmaeildoost *et al.*, 2021 ▸). We remark that the shape of the second-order component identified in the present study is highly similar to the first-order component identified in very recent work on impulsive heating of deeply supercooled water at *T* = 230 K (Tyburski *et al.*, 2026 ▸).

Summarizing the sections above, we have here presented a set of experiments focused on the changes in X-ray scattering from pure water as a function of temperature. The shape of the difference signal(s) Δ*S*(*Q*, Δ*T*) describing these changes have been found to be in very good agreement with previous results based on time-resolved studies at ESRF. However, we find a significant discrepancy of ∼30% in the magnitude of these difference signals, with the magnitude of the ESRF-determined difference signal, 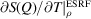, likely being too low. Further analysis based on a Taylor expansion allows the determination of both first- and second-order components of the difference scattering signal with a very good signal-to-noise ratio, and the results of this analysis have been archived for community use. While the new reference data can be utilized for analysis as-is, we note that any particular application will require interpolation onto the experimental *Q*-axis, including possibly some convolution/broadening if data were acquired with a more polychromatic beam than used to acquire this new reference signal. For experiments with a significantly different *Q*-axis coverage or at a different temperature range, incorporating a measurement of ∂*S*(*Q*)/∂*T*|_ρ_ in the experimental protocol is advised, with subsequent scaling of the magnitude of the experiment-specific difference signal to the reference provided by the present work.

## Supplementary Material

Supplentary information including figures. DOI: 10.1107/S160057752600250X/mad5003sup1.pdf

## Data Availability

The reference data and data analysis script are available at DTU Data at https://doi.org/10.11583/DTU.c.8315629.
