# Supplementary material for: A new reference for changes in X-ray scattering from water due to temperature changes at ambient conditions
Source: J Synchrotron Radiat. 2026 Apr 14;33(Pt 3):649–57. doi: 10.1107/S160057752600250X (PMC13148597; doi:10.1107/S160057752600250X)
Supplement: Supplementary file 1 [file s-33-00649-sup1.pdf]

JOURNAL OF SYNCHROTRON RADIATION

Volume 33 (2026)

Supporting information for article:

A new reference for changes in X-ray scattering from water due to temperature changes at ambient conditions

Lise G. Hanson, Thomas Veile, Jonathan F. Schouenborg, Victor M. Nielsen, Mads R. V. Jørgensen, Frederik H. Gjørup, Cathrine Frandsen and Kristoffer Haldrup

A New Reference for Changes in X-ray Scattering from Water due to Temperature Changes at Ambient Conditions

March 6, 2026

Lise G. Hanson [a], Thomas Veile [a], Jonathan F. Schouenborg [a], Victor M. Nielsen [a], Mads R. V. Jørgensen [b], Frederik H. Gjørup [b], Cathrine Frandsen [a], Kristoffer Haldrup [†] [a]

[a]Department of Physics, Technical University of Denmark, 2800 Kongens Lyngby, Denmark
[b]Department of Chemistry and iNANO, Aarhus University, Langelandsgade, Aarhus, 8000, Denmark & MAX IV Laboratory, Lund University, Fotongatan 2, 221 00, Lund, Sweden
[†]Corresponding Author

SI 1 Background subtraction and conversion to electron units

In this work, the background subtraction and conversion of the intensity to electron units per H_2O molecule have been obtained by scaling the data to a reference signal $S(Q)_{\mathrm{H_2O,\ ref.}}$ measured by (Skinner *et al.*, 2013). This reference signal is measured on water at 295 K, and is given in units of e.u./H_2O.

The first step is to subtract the background scattering (capillary and air) from the measured signal $S(Q)_{\mathrm{Meas.}}$ originating from the sample (combination of water, capillary, and air). The background subtraction is done by fitting the following expression

$$S(Q, 300\ \mathrm{K})_{\mathrm{Meas.}} = \alpha\, S(Q)_{\mathrm{Bg.}} + \beta\, S(Q)_{\mathrm{H_2O,\ ref.}}, \tag{SI 1}$$

where $S(Q)_{\mathrm{Bg.}}$ is the measured intensity from an empty capillary (scattering from capillary and air). $S(Q)_{\mathrm{Bg.}}$ is taken as an average over all temperatures (ramp from 300 K to 330 K) to obtain better measurement statistics. This averaging is possible since the scattering of the quartz capillary changes insignificantly with temperature, see Section SI 2. α and β are fitting parameters with α determining the amount of background signal relative to the water signal. α is determined from $S(Q, 300\ \mathrm{K})_{\mathrm{Meas.}}$ since 300 K is the temperature closest to the 295 K at which $S(Q)_{\mathrm{H_2O,\ ref.}}$ is measured. After the value of α has been determined, the background is subtracted to determine the corrected water signal

$$S(Q, T)_{\mathrm{H_2O,\ corr.}} = S(Q, T)_{\mathrm{Meas.}} - \alpha S(Q)_{\mathrm{Bg.}} \tag{SI 2}$$

Hereafter, the intensity is normalized into e.u./H_2O by normalising with the integral of the reference

signal

$$S(Q,T)_{\mathrm{H_2O,\ scaled}} = S(Q,T)_{\mathrm{H_2O,\ corr.}} \frac{\int S(Q)_{\mathrm{H_2O,\ ref.}}\, dQ}{\int S(Q,T)_{\mathrm{H_2O,\ corr.}}\, dQ}\,. \tag{SI 3}$$

Figure SI 1 shows $S(Q,T)_{\mathrm{H_2O,\ scaled}}$, which is called $S(Q,T)$ for simplicity, and compare it with $S(Q)_{\mathrm{H_2O,\ ref.}}$.

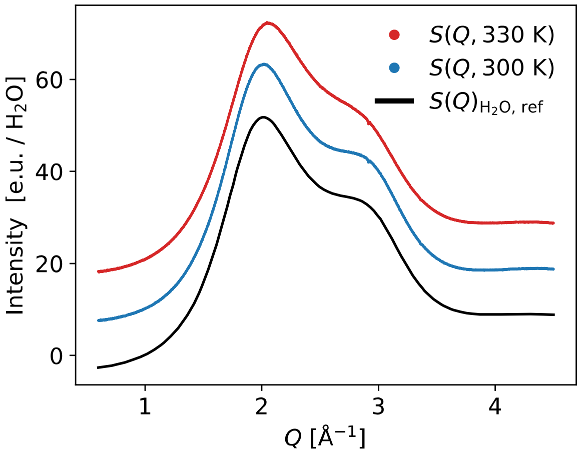

Figure SI 1: Comparison of the reference signal of water, $S(Q)_{\mathrm{H_2O,\ ref.}}$, by Skinner *et al.*, 2013 (black curve) with the scattering data $S(Q,T)$ measured in this work at DanMAX (red and blue) after background subtraction and conversion to electron units. An offset is used for clarity.

SI 2 Changes in capillary scattering due to temperature

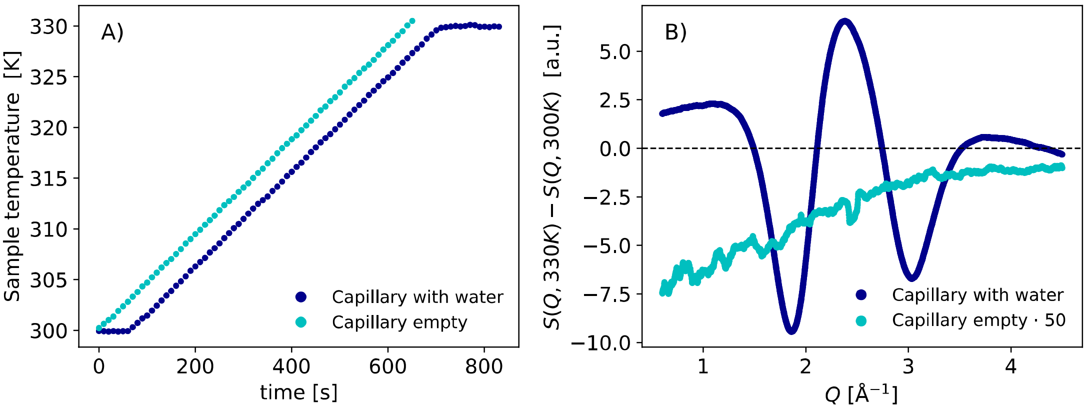

Figure SI 2: **A)** Temperature evolution of the sample with water in a capillary and an empty capillary. In the measurement on a capillary with water (dark blue), steady state measurements at 300 K and 330 K were performed before and after the temperature ramp, respectively. **B)** Difference scattering of water and the empty capillary across $\Delta T = 30$ K. The difference scattering of the capillary is multiplied by 50 for clarity of the plot. The data is smoothed by a mean filter of width 0.08 $Å^{-1}$.

Here it is investigated how much the capillary scattering changes with temperature relative to the difference scattering from water. Figure SI 2A shows the temperature evolution of the sample capillary with water and the empty capillary, where the temperature in both cases is ramped linearly between 300 K and 330 K. Figure SI 2B shows the difference scattering $\Delta S = S(330\text{ K}) - S(300\text{ K})$ for the sample capillary with water and the empty capillary. Notice that the difference scattering of the empty capillary is multiplied by 50. It is seen that the difference scattering of the quartz capillary is insignificant relative to the difference scattering of the water. Thus, for the background subtraction, it was decided to use the time average of the empty capillary to get better statistics. Taking the time average is justified by the low change in the difference scattering in Figure SI 2B of an empty capillary relative to a capillary with water.

SI 3 Noise and resolution from steady-state measurements

The uncertainty of $\Delta S(Q)$ is determined from the standard deviation of the difference data at steady state, see Figure SI 3. The standard deviation must be calculated at steady state, as any changes in temperature would cause a change in ΔS, which is not caused by noise but caused by real signal changes.

To calculate noise at steady state, the signal differences are calculated between consecutive data

points

$$\delta S(Q) = S(Q)_i - S(Q)_{i-1} \tag{SI 4}$$

where the subscript is the index of the data points. The uncertainty of $\Delta S(Q)$ is taken as the standard deviation of $\delta S(Q)$. Figure SI 3 shows the uncertainty measured at steady state at 300 K and 330 K. It is seen that the standard deviation does not change with temperature, but it varies with Q, which is to be expected as the signal noise scales with $\sqrt{S}$. The standard deviation is taken to be the one measured for 330 K (black) because more data points were available at the high temperature steady state, giving a more accurate estimate of the uncertainty. The uncertainty is on the order of 0.05 e.u., while ΔS at ΔT = 3K is on the order of 0.3 e.u corresponding to a relative uncertainty around 17 %.

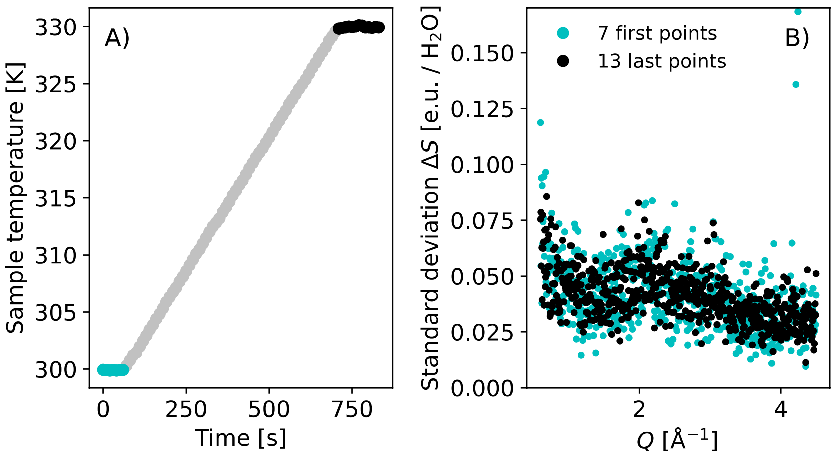

Figure SI 3: **A)** Temperature of the capillary with water. The black and blue regions indicate where the standard deviation is determined. **B)** Standard deviations of $\delta S(Q)$ determined from the steady state regions at 300 K (cyan) and 330 K (black).

SI 4 Q-correction for ESRF ID09B reference data

To ensure full comparability between the data acquired in 2011 at ESRF and in 2024 at MaxIV, it was checked whether the Q-scales were exactly similar as this may change due to even small errors in the sample-detector distance. Equation SI 5 below shows the correction which should applied to the Q-axis if an offset in the sample-detector distance is needed. Assuming the MaxIV data to be correct due to the accurate LaB_6 calibration procedure applied, Figure SI 4 shows the best-fit value determined when fitting the $\Delta S(Q)^{\mathrm{ESRF}}$ to $\Delta S(Q)^{\mathrm{MaxIV}}$ as a function of the offset (as well as ΔT and $\Delta\rho$). After fitting ΔT, $\Delta\rho$, and x for all values of T_1, the average sample detector distance correction is used as a fixed correction value to calculate Q_{new} in the rest of the analysis. After having corrected Q into Q_{new}, the fits of ΔT and $\Delta\rho$ are always determined without fitting

x.

To correct the Q-values from the originally reported values, Q_{old}, into the new ones, Q_{new} expected from a relative sample displacement x we follow the procedure

$$Q_{\text{new}} = \text{Re}\left\{\frac{4\pi}{\lambda}\sqrt{\frac{1}{2}\left(1 - \frac{1}{1+\text{var}_2}\right)}\right\} \tag{SI 5}$$

$$\text{var}_2 = \left(\frac{1}{1+x}\right)^2 \left[\left(\frac{1}{1-\text{var}_1/8}\right)^2 - 1\right] \tag{SI 6}$$

$$\text{var}_1 = \left(\frac{\lambda Q_{\text{old}}}{\pi}\right)^2 \tag{SI 7}$$

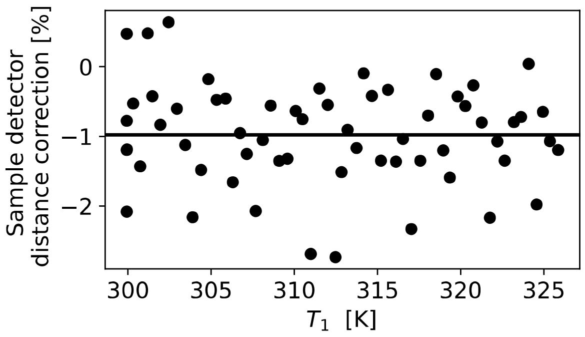

Figure SI 4: Fitted value of the sample detector distance displacement percentage, x.

SI 5 Density contribution

Density data on Water at 1 atm has been found from the NIST Chemistry WebBook, SRD 69 Thermophysical Properties of Fluid Systems (Lemmon, 2010). The density data used in this study are shown in Figure SI 5A. The expected change in density, $\Delta\rho_{1\ \text{atm}}$, as a function of temperature is calculated from the NIST data by

$$\Delta\rho_{1\ \text{atm}}(T_1) = \rho_{\text{NIST}}(T_1 + \Delta T) - \rho_{\text{NIST}}(T_1). \tag{SI 8}$$

Figure SI 5B shows the values of $\Delta\rho_{1\ \text{atm}}(T_1)$ with $\Delta T = 3$ K maintained fixed.

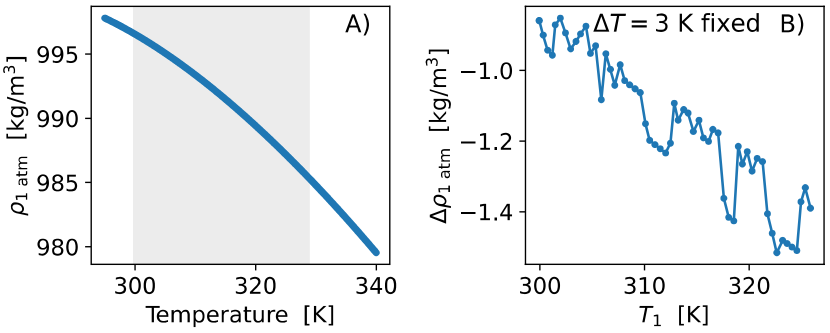

Figure SI 5: **A)** Density of water as a function of temperature at 1 atm. The data is found from (Lemmon, 2010). The gray box indicates the region from which the theoretical values of $\Delta\rho_{1\ \mathrm{atm}}$ is calculated. **B)** Density change, $\Delta\rho_{1\ \mathrm{atm}}$, calculated from the data shown to the left (gray region) given a fixed temperature change of $\Delta T = 3$ K. The $\Delta\rho_{1\ \mathrm{atm}}$ data is the same as shown in Figure 4.

SI 6 Investigation of difference scattering with ΔT=3,7 or 15 K

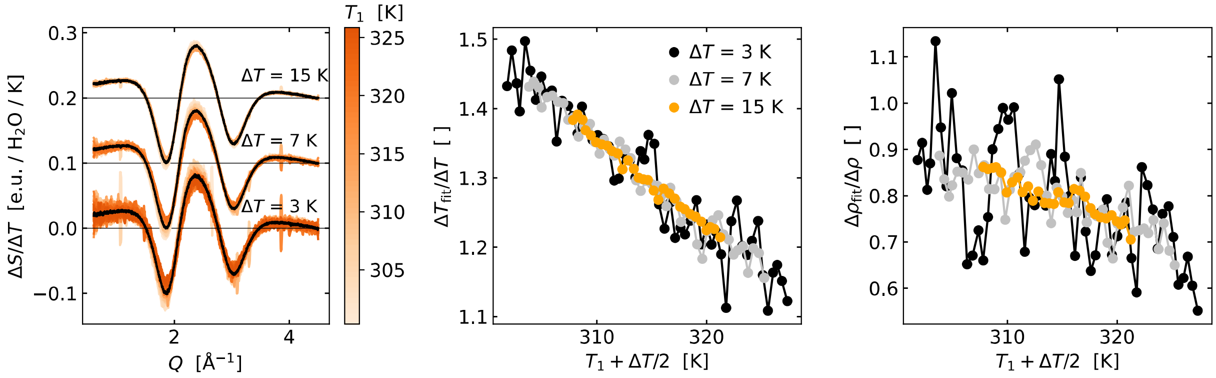

Figure SI 6: Difference signals, fits and residuals for $\Delta T = 3$, 7, and 15 K. **A)** $\Delta S/\Delta T$ for all three values of ΔT. The orange curves show $\Delta S/\Delta T$ for different values of T_1 and the black curve shows the average of $\Delta S/\Delta T$ across all T_1. An offset is used for clarity. **B)** Results for ΔT_{fit} when using the three different values of ΔT. The y-axis gives the central temperature of the fitting region. **C)** Results for $\Delta\rho_{\mathrm{fit}}$ when using the three different values of ΔT.

An analysis identical to the one performed in Figure 3B and 4 is performed in Figure SI 6 with $\Delta T = 3$, 7, and 15 K. From comparing the results across different ΔT values it is seen that the fitting values ΔT_{fit} and $\Delta\rho_{\mathrm{fit}}$ are not affected by the choice of ΔT used in the analysis. Thus, the non-linearity observed in Figure 4 is not an effect caused by the value of ΔT used in the data analysis. In the article, $\Delta T = 3$ K has primarily been used.

SI 7 Temperature calibration

Figure SI 7 shows a photo of the experimental setup where a capillary with water is heated by a heat gun while the capillary is rotated by a capillary spinner. The heating power in the heat gun is controlled by a thermocouple, which is permanently sitting in the heat gun nozzle. The sample is rotated to ensure a homogeneous temperature distribution (heating from all sides and mixing of fluid) and a precise sample detector distance.

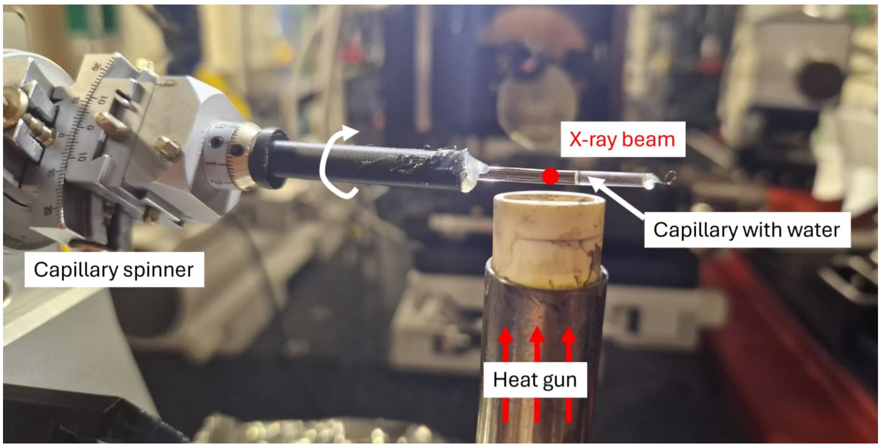

Figure SI 7: Photo of the setup with the heat gun heating the sample capillary and a capillary spinner, which rotates the sample.

To know the temperature at the sample position, a calibration measurement was performed to relate the temperature measured inside the heat gun nozzle to the temperature at the sample position. In the calibration measurement, a thermocouple was placed at the sample position instead of having a sample capillary. The tip of the sample thermocouple was carefully positioned at the same position as the sample capillary since the temperature varies with position relative to the heat gun (most strongly with height over the heat gun). A laser beam travelling along the X-ray beam was used to help obtain a precise thermocouple position in the vertical and left-right direction in the picture seen in Figure SI 7.

Figure SI 8A shows the temperature measured at the sample position as a function of the temperature measured in the heat gun nozzle. A second-order polynomial is fitted to the temperature data, and this fit serves as the calibration between the two temperatures (nozzle and sample). Figure SI 8B shows the residual of the fit. A good agreement is seen as the residual from the fit has a maximum of 0.3°C, and the standard deviation is 0.07°C. There are some systematic deviations in the residuals, but since these are small, it is considered unnecessary to correct for them.

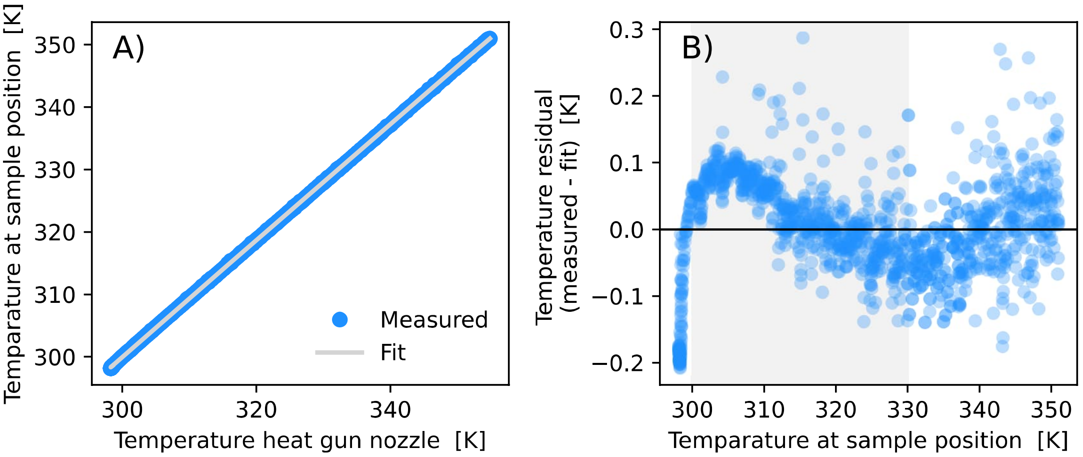

Figure SI 8: **A)** The temperature measured at the sample position vs. the temperature measured inside the heat gun nozzle. Both temperatures are measured by thermocouples, and the nozzle temperature is used to control the heating power in the heat gun. A second-order polynomial is fitted to the temperature data. This fit is used to calculate temperatures in the samples based on the nozzle temperature. **B)** Temperature residual between the measured temperature at the sample position and the fitted value. The grey area indicates the temperature region used in this study.

608 The calibration function is determined to

$$T_{\mathrm{sample}} = 2.50\ \mathrm{K} + 1.04\, T_{\mathrm{nozzle}} - 0.000175\ \mathrm{K}^{-1}\, T_{\mathrm{nozzle}}^2 \tag{SI 9}$$

609 SI 8 Comparison of the reference signals

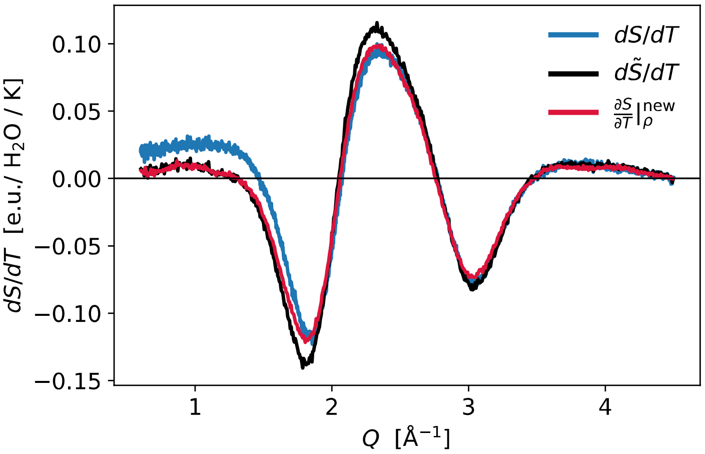

Figure SI 9: Comparison of the first-order derivatives reference signals.

Figure SI 9 shows the first-order derivative parts of the three new reference signals determined from this study. The blue and black curves are different in scaling and shape since only the black curve is corrected for the density contribution. Both the blue and black signals evaluate the derivative at 300 K due to the Taylor expansion method. The red curve ($\frac{\partial S}{\partial T}|_{\rho}^{\text{New}}$) is different from the two other signals because this reference signal does not take into account the second-order contribution. The red curve is calculated from an average across the 300-326 K region (see equation 3 in the main article) and is thus most accurate in the middle of this region, i.e., at 313 K. It is observed that the scalings are different for the red ($\frac{\partial S}{\partial T}|_{\rho}^{\text{New}}$) and black $\frac{d\tilde{S}}{dT}$ curves, while their shapes are very similar, see Figure SI 10 also to that of $\frac{\partial S}{\partial T}|_{\rho}^{\text{ESRF}}$.

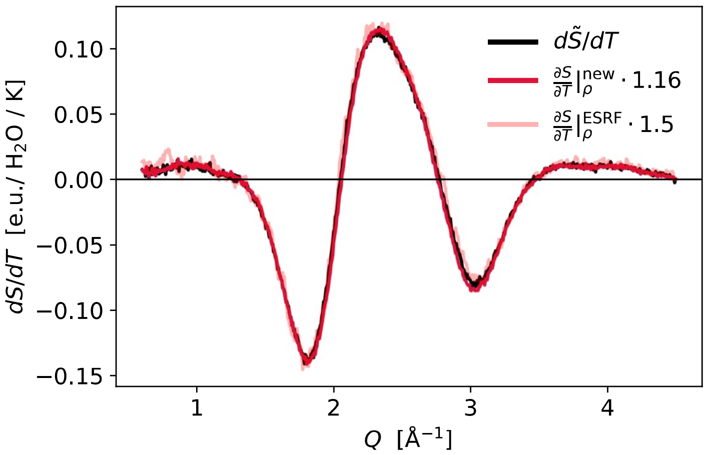

Figure SI 10: Comparison of the three different first-order derivative reference signals scaled such they match in height with $d\tilde{S}/dT$.

The lower magnitude of $\frac{\partial S}{\partial T}|_{\rho}^{\text{New}}$ relative to $\frac{d\tilde{S}}{dT}$ can be understood from Figure SI 11, which shows the values of $\frac{\partial S}{\partial T}|_{\rho}^{\text{New}}$ for different values of T_1 when no averaging is performed i.e.,

$$\left.\frac{\partial S(Q,T_1)}{\partial T}\right|_{\rho}^{\text{New}} = \frac{\Delta S(Q,T_1)_{\Delta T}}{\Delta T}\,. \qquad \text{(SI 10)}$$

From Figure SI 11 it is clearly seen that the signal decreases with T_1 and thus the mean $\frac{\partial S}{\partial T}|_{\rho}^{\text{New}}$-value at $\langle T_1 \rangle = 313$ K will be lower than if the $\frac{\partial S}{\partial T}|_{\rho}^{\text{New}}$ signal was calculated only at 300 K.

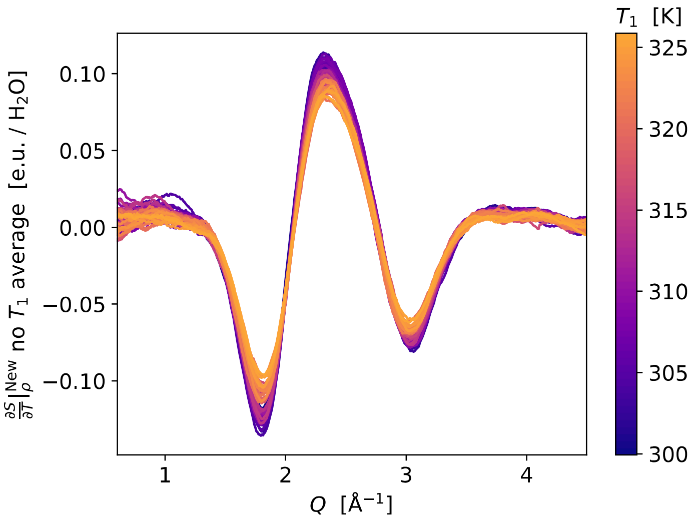

Figure SI 11: Value of $\frac{\partial S}{\partial T}|_\rho^{\mathrm{New}}$ if not averaged over T_1, i.e., Equation SI 10.

In principle, $\frac{\partial S}{\partial T}|_\rho^{\mathrm{New}}$ could also be used as the new reference signal. $\frac{\partial S}{\partial T}|_\rho^{\mathrm{New}}$ signal is valid in the temperature region 300 K to 326 K, with expected changes in magnitude caused by the non-linearity of up to ±8% at 300 K and 326 K, respectively. $\frac{\partial S}{\partial T}|_\rho^{\mathrm{New}}$ has the highest validity at the centre of the measured temperature region, i.e., 313 K, due to the averaging in Equation 3.
